# The Role of Universities in Social Innovation Within Quadruple/Quintuple Helix Model: Practical Implications from Polish Experience

**DOI:** 10.1007/s13132-021-00804-y

**Published:** 2021-06-20

**Authors:** Joanna Morawska-Jancelewicz

**Affiliations:** Faculty of Human Geography and Planning, Adam Mickiewicz University, Poznan, Poland

**Keywords:** Quadruple/quintuple helix model, Fourth mission, Socially engaged university

## Abstract

The social dimension of innovation is growing due to unprecedented wicked global challenges we all face, including COVID-19 pandemic. Those challenges in their dynamic complexity require new cross-scale, cross-domain and action-oriented approaches at the universities. I argue that universities need to go beyond their traditional missions and to take an active role in a transformative change by working with their communities and creating real social impact. The fourth mission concept is particularly relevant as it puts emphasis on the universities roles in sustainable development. I propose to reflect on those new roles of universities in the context of quadruple/quintuple helix model that is seen as playing an important role in fostering the shift from technical to social innovations. Social innovation is an element of a regional innovation system, in which the importance of knowledge is not determined exclusively by competitiveness and productivity, but by taking into account the creation of social well-being, the impact on the quality of life and co-creation of knowledge as part of public–private partnerships. By addressing social innovation practices from a perspective of Polish public universities, it fills the gap of relatively few studies on institutional change and incentive structures that influences the ability of universities to engage in social innovation by proposing. I propose to adapt the socially engaged university model that could be a tool for stimulating and strengthening their functions within a modern regional innovation system allowing for an active role of civil society organisations.

Innovation is not identical with social well-being, but social well-being may be achieved faster thanks to innovation… provided the social objective is at the heart of innovation.^1^

## Introduction

The present global challenges, exemplified in the United Nations Development Program ‘Sustainable Development Goals’, affect regional innovation systems that need to develop new approaches, new forms of collective actions between public and private stakeholders, as well as new solutions with the aim to address social challenges through innovation. Those challenges cannot be simply addressed from a unilateral perspective, and social innovation is recognised as an important component of this new framework. Thus, there is a need to find ways to foster innovation which generates social and public value (OECD, 2011). The COVID-19 crisis has pushed organisations, including universities, to test new ways of stimulating social innovation. The recently discussed concept of Society 5.0 and Industry 5.0 (Carayannis et al., 2021; Carayannis et al., 2020) highlights the need to re-think existing working methods and approaches toward innovation and to focus them on developing human-oriented solutions and social innovation.

Undoubtedly, innovation policy must be confronted with the pressure of ongoing changes, what Edquist called broad-based innovation (2009), that encompasses new forms of innovation like open innovation (Chesbrough, 2003), lead user innovation (Hippel, 2005), user-oriented innovation (Arnkil et al., 2010), and social innovation (Adams & Hess, 2010; Klein, 2017; Mulgan, 2007, 2010; Moulaert, 2009; Murray et al., 2010). They are based on the active role of citizens and civil society organisations in the innovation process. This has a direct impact on innovation systems that have become not linear, network-based, and deeply rooted in a regional context (McAdam et al., 2016). Sources of innovation are no longer restricted to interactions between university-industry government in the traditional triple helix model of innovation (Etzkovitz & Leydesdorff, 1997). They become more heterogenous and socially distributed. The quadruple helix/quintuple helix model (QHM) expands the triple helix by adding the fourth dimension, that is civil society and the environment (Arnkil et al., 2010; Carayannis & Campbell, 2012; Carayannis & Grigoroudis, 2016; Carayannis & Rakhmatullin, 2014). Civil society is understood as ‘non-governmental organisations, as well as more or less formal associations and communities of interest and practice including engaging citizens as lead-users, co-developers and co-creators of innovative and entrepreneurial initiatives’ (Carayannis et al., 2019). This model relies on the understanding that additional perspectives must be added to comprehend innovation in the unfolding twenty-first century. This concept allows to integrate in the system a bottom-up approach (complementing the top-down policies and practices) and form a more inclusive, democratic system based on a dialogue and reflecting also values of the society. ‘In fact, democracy frames and changes our conditions of innovation’ (Cavallini et al., 2016, p. 14). A new approach is necessary to solve problems where social and technological progress co-evolves in order to generate social value.

In this new paradigm, the importance of knowledge is not determined exclusively by competitiveness and productivity, but by taking into account the creation of social well-being, the impact on the quality of life and co-creation of knowledge as part of public–private partnerships. The existence of a well-developed network in a given territory makes it possible to combine and strengthen the actions of all entities (actors), which influences, as a result, the acquisition of a collective skill conducive to innovation processes. As Trencher et al. indicate (2013, p. 40), ‘the global transition to a sustainable society will ultimately be the sum of a decentralised transformation carried out by countless individual communities and regions across the planet that could significantly contribute to a local or regional transition to sustainability’. This represents a shift from the idea of merely contributing to economic and societal development via technology transfer and entrepreneurialism, to collaborating with diverse external actors to create societal transformations in view of materialising sustainable development.

Existing global challenges and rapid technological progress make functioning in today’s world increasingly complex and complicated, and have led to growing expectations towards universities and their roles in modern ecosystems. Responding to sustainability challenges requires not only trans- and multi-disciplinary approaches but also high level of engagement of social and human capital. Social dimension of innovation is growing and results therefore from occurring social and economic changes for which new solutions are being sought. Today, universities are expected to fulfil manifold and increasingly challenging roles. Finding balance between participation in solving global issues and their local contexts is one of the challenges for the academic environment today. Different approaches and models are investigated that reflect those new university roles. They rely on the multi-stakeholder initiatives. As Dentoni and Bitzer argue (2014), they can be seen as a response to the increasing urgency of many wicked problems that call for transdisciplinary responses and collective actions. It is generally recognised that if universities wish to actively contribute to sustainability they need to go beyond their traditional functions of education, research and community outreach and to integrate social innovation in their core and new missions (Bayuno et al., 2020). A renewed EU Agenda for Higher Education (2017) emphasise ‘HEIs should be engaged in the development of their cities and regions, whether through contributing to development strategies, cooperation with businesses, the public and voluntary sectors or supporting public dialogue about societal issues. Outreach beyond the academic community in local languages should be incentivised and rewarded, including as part of career development’.

Social innovation is well conceptualised and developed in literature, but less attention is given to universities as agents of change. The focus is put rather on civil society organisations or social entrepreneurs rather than research organisations and universities. As Bayuno et al. argue (2020, p. 2), ‘relatively few studies address issues related to institutional change and incentive structures that influences the ability of universities to engage in social innovation’. This argument is also in line with what Benneworth et al. (2020, p. 32): ‘although universities have a huge potential to contribute their knowledge and other assets to social innovation, a recent inventory of social innovation in Europe highlighted how underdeveloped and one-dimensional these contributions were’. This is first knowledge gap I aim to address in this paper by proposing of socially engaged university model focused on social innovation and universities internal dynamics and their core missions. However, the external dynamics should also be investigated given that the new paradigm of knowledge democratisation is build upon the cooperation with non-academic actors. Surely, ‘universities are complicated mixtures of different communities with changing power and specific relations with external actors’ (Arocena & Stutz, 2021, p. 4). Those new types of relations are reflected in QHM and ‘only few contributions have explored the connection between the social innovation concept and the QHM framework’ (Bellandi et al., 2021, p. 8). In this paper, I try to address this gap and argue that, apart from differences between universities (in terms of their history, relevance, missions, profile, research and education strategies, funding, etc.), their embeddedness in regional ecosystem of innovation is one of key dimensions that can influence their engagement in social innovation. I also argue in this paper that social innovation should be extended to all the missions and I try to overcome the dominating narrative in literature that concentrate on discussing this concept only through the third mission/community engagement concept. Still I acknowledge after Bayuno et al. (2020, p. 8) ‘that further research on how universities can benefit directly from social innovations teaching and research may entice universities to commit adequate resources to it’. Since the connection between QHM and social innovation is still in infancy (Bellandi et al., 2021, p. 5) I try to approach this concern through a case study of Polish public universities experience and by this I try to address the contribution of universities to social innovation.

The literature analysis on the role of Polish universities in the process of social innovation pointed to a small number of empirical studies and relatively poor theoretical and source material covering this sphere of Polish universities activity. In fact, only one comprehensive work was available at the commencement of this study (Baran, 2016) that addressed the engagement of Central European universities (including Polish universities) in matters of social innovation. I refer to this work in a discussion and conclusions. This paper, however, attempts not only to analyse the existing experience, policies and practices of Polish universities in social innovation but also to address the need for more systemic approach resulting from the ongoing dynamic innovation processes. It tries to embed the social innovation in a broader context of new roles of universities in quadruple helix. My research focuses not only on internal dimensions of engaging in social innovation by universities but it addresses the ongoing global debate on the social and sustainable challenges and the responding new cooperative models in innovation systems. The main purpose of this article is to contribute to university policy and practice in implementing social innovation in a collaborative process. Specific research questions are the following: (1) What conditions must be met for universities to systematically support and/or generate social innovation? (2) What measures should universities take to stimulate and strengthen their functions within the QHM, i.e. a modern regional innovation system allowing for an active role of civil society organisations? (3) What are the key features of the socially engaged university model that contributes to social innovation in a systemic way? To address these questions, I conducted an empirical study based on a sample of 63 Polish public universities. As far as the practical aim is concerned, the paper proposes a model structure of a socially responsible and socially engaged university within QHM focusing on social innovation but extending far beyond and contributing to the development of the strategic approach to universities’ social missions and impacts in modern economies and societies. The starting point for these considerations is the assumption that.

*if universities want to build an effective system of cooperation with the social and business environment, they should create proper structures and mechanisms supporting the development and implementation of social innovations in a region, thus contributing to the practical application of university social responsibility.*

The organisation of the paper is designed in the following way. The theoretical part (section ‘Modern Processes of Innovation’, ‘The Growing Importance of Social Innovation’, ‘New Approaches to Universities Missions and Their Role in Social Innovation’) refers to the results obtained from the literature review on modern theories of regional growth, regional innovation systems, social innovation dimensions and definitions of social innovation. It also explains the specificity of university activity in the context of cooperation with its stakeholders and identifies the main directions of the development of the so-called third and fourth mission of universities and the role they play in regional and local networks of knowledge. Apart from scientific papers, the results of the chosen projects (e.g. IMPACT, SIC Europe, SI-DRIVE, TEPSI), as well as reports prepared by national and foreign organisations (NESTA, The Young Foundation, OECD, the European Commission), were an inspiration for this paper. They cover mainly the experience of Europe and North America.

The empirical part (section ‘Research on the Potential of Polish Universities Regarding the Implementation of Social Innovations’, ‘Summary of Research Results’) concerns the conducted analysis of the use of the potential of Polish universities regarding the implementation of social innovations and contains an attempt to determine factors influencing their growth. I propose the conception of a model of a socially engaged university, the activity of which contributes to supporting the regional innovation system and to creating and implementing social innovations. The research results and the conclusions drawn therefrom may add value to the ongoing scientific discourse on the development of social innovation by universities and their importance for the innovative growth of regions within QHM.

## Modern Processes of Innovation

In the late twentieth century, there was a methodological revaluation in regional research due to an increasing discrepancy between trends in the development of theoretical conceptions and real changes occurring in the world. Not only did technological progress fail to eliminate differences in the regional development level, but it increased them, and was therefore not a sufficient variable to explain significant and widening regional differences.

The complexity and multidimensionality of innovation processes result from two trends in economic thought. The first rests on evolutionary theories of economic and technological changes, exposing the evolutionary and systemic character of the innovation process in a specific institutional context. In this approach, innovation is a process stemming from the evolution of institutions ‘attached’ to the development path of a given territory. The second trend sees innovation as an interactive, social process, created and developed by many participants, dependent on many factors, including the collective learning process of entities and institutions linked by networks of dependencies. Knowledge is treated as a fundamental resource, on which socio-economic growth is based, leading to the development of a knowledge-based economy (Etzkovitz & Leydesdorff, 1997, Florida, 2004, Foray, 2014, Kahin & Foray, 2006, Nowakowska, 2011, OECD & World Bank, 2000, Powell & Snellman, 2004). Innovativeness, understood as the ability to make changes in the economy that cause positive technical–economic or social results and producing beneficial economic effects, is a necessary condition for the development of a region (Gaczek, 2009).

Modern innovation processes depend largely on nontechnological factors and are network and systemic in nature. They are created by many interdependent entities and require access to various resources (knowledge, information and competence). The existence of a network or an innovation system on a given territory makes it possible to combine and strengthen the activities of all entities, which results in the acquisition of a collective skill conducive to innovation processes. Innovation has its source in various social and economic institutions interlinked by a coherent technological infrastructure. This is a derivative of interactions brought by the cooperation of many actors and is the effect of synergic and collective action, not an individual activity. Contemporary innovation models are therefore network and systemic in nature, in contrast to earlier unidirectional, linear or sequential models (Nowakowska, 2011). Hence, the importance of reflections about the significance of social capital and its relation to human capital in research on regional development. The change in the approach to creating innovation systems and the shift of the focus from the triple helix model to the idea of including civic society in the innovation process results, among other things, from social changes and accompanying global transformations which require a different approach. A triple helix model is insufficient to understand a long-term innovation process which requires that cooperation between all actors be taken into account, including social partners and civic society organisations. The limitations of the triple helix model result also from the insufficient consideration of changes which occurred in the area of new technologies, such as nanotechnology or biotechnology, ICT. The division of science into disciplines ceases to be the only model of organising research. The creation of new knowledge is increasingly interdisciplinary, non-linear, comprehensive, and often hybrid. Moreover, the inclusion of the fourth and fifth helixes in the model seems to be crucial because the usefulness of knowledge is more and more often verified in terms of its social value. Public good and social wellbeing is of key importance here.

The quadruple helix model is seen as playing an important role in fostering the shift from technical to social innovations (Carayannis & Rakhmatullin, 2014), though civil society participation in the context of regional innovation systems continues to be low, and the regions have experienced difficulties in getting civil society groups involved (Roman et al., 2020). This is a model of innovative cooperation or an innovative environment, in which innovation users (citizens), companies, universities and public authorities cooperate to generate socially significant innovations. QHM helps to strengthen knowledge-based society and knowledge-based democracy. In addition to innovation activity linked to the high-tech sector, this model also allows for innovations created directly by recipients and provides different variants of knowledge and technology applications (Arnkil et al., 2010). Roman et al. (2020) highlight that citizens can have the power to suggest new types of innovation. Users, i.e. citizens, become the driving force behind the innovation process, both at the design stage and during the implementation. Benneworth et al. (2020) stress that the basis of the QHM is that social systems and actors also participate in collaborative interactions with other kinds of knowledge actors to shape development trajectories and stimulate innovation. The role of the latter is particularly important because users of innovation actively participate in the creation thereof. Socio-economic development is created through continuous innovation between four helices (Alfonso et al., 2012). The fourth helix allows for the meaning of new founding and innovations which are to improve the quality of life and strengthen social well-being. This model helps therefore to establish ties and relations between participants of the innovation process. It also affects the creation of scientific and education development strategies responding to contemporary challenges (Arnkil et al., 2010). The QHM is flexible and may be extended or modified taking account of contemporary challenges and problems. The need for a more sustainable development and climate change challenges are reflected in quintuple helix model (Carayannis & Campbell, 2012; Carayannis et al., 2019) that adds the fifth dimension—the environment—and sets the stage for sustainability priorities and considerations so that nature is central and equivalent component of and for knowledge production and innovation. It becomes recognised as an essential and equal element in the innovation and knowledge system (Carayannis et al., 2019). The QHM helps to include society in the innovation system, which leads to the emergence of new innovation forms and a new way of organising networks between various interested parties. What stands out in the research on innovation processes is the conception of open innovation, proposed by Chesbrough (2003), which assumes that research and development studies conducted in a company can be inspired by good ideas generated either inside or outside a given organisation and may reach the market through or outside the company. These external ‘paths’ are as important as internal ones (Arnkil et al., 2010). Hippel (2005) proposed a similar conception, based on a so-called lead user, who can be either a company or a person. This conception considers it crucial to know the needs, expectations and tastes of consumers, to use their ideas and to involve them in the creation of innovations. It assumes that users often create better solutions which meet their unique needs. This approach leads to social innovation.

## The Growing Importance of Social Innovation

The existing literature explores social innovation from a wide range of perspectives. Social innovation has been recognised as the solution to modern problems (Anderson et al., 2018; Klein, 2017; Moulaert et al., 2017; Baran, 2016; Benneworth & Cunha, 2015; Boelman et al., 2014, Wallace, 2017). The European Union supports its development and highlights the importance of cooperation between science and society for solving social problems, thorough e.g. the Horizon 2020 program *Science with and for Society* and new concept of *EU Missions*. It also promotes the idea of combining scientific excellence with awareness and social responsibility. Social aspects of innovation are also considered in planning urban and regional development. A prosocial trend can be also indicated in the programming of selected national and EU policies. Social innovation is based on the assumptions of open innovations and is part of the conception of regional endogenous development, where a key factor of development is people with their knowledge and skills. The progress is assumed to be endogenous in character and the role of a particular factor leading to an increase in the productivity of other resources is ascribed to human capital and knowledge. This conception emphasises the noneconomic nature of development factors. It also points out that innovations result to a great extent from the accumulation of the experiences of a given actor (mainly through learning by doing) and from continuous learning through interaction (learning by interaction). This leads to an improvement in skills and qualifications, and contributes to increased labour productivity and technological advancement. The accumulation of knowledge in a specific space, along with learning mechanisms makes it possible to create and disseminate new knowledge resources and innovation processes (Gaczek, 2009; Nowakowska, 2011). Adams and Hess (2010) notice that local communities and interested parties are drivers of innovative changes. This is typical of new types of innovations, including social ones. Innovations appear as a result of cooperative activities, and networks, clusters or common values and interests are keys to their creation.

Social innovations are commonly known as ‘innovations with a human face’. They are often understood as activities undertaken by citizens and urban activists and related to excluded groups. However, social innovations represent a very wide range of activities, embrace various sectors, fit into a wide spectrum of scientific disciplines, they are developed and proposed by various types of organisations, interested parties, teams or individuals. Advanced IT tools and new technologies are increasingly often used to implement them. The priority objective of social innovations is social change. They are determined by an additional motive, i.e. a social mission and social added value. It does not mean depriving them of economic viability. Yet, the underlying purpose of innovative ideas should be the solution to a specific problem and a change for the better. They often require an interdisciplinary approach combining frequently remote fields of knowledge and are created by teams of people representing different organisations and competences.

As Mihci (2019) and Bayuno et al. (2020) point out, it is difficult to reach a common acceptable definition for social innovation. Moreover, there is little clarity about the actual meaning of social innovation, and there is even no consensus at all regarding its relevance in the social sciences accordingly, some even go further to argue that the term should be abandoned as a scientific concept. Social innovations are a qualitatively new element in the process of socio-economic evolutionary development based on knowledge and innovation. Both the content given to the concept of social innovation and its functioning and development reflect the complexity of the socio-economic processes of the early twenty-first century. There are not only numerous definition concepts but also a number of different classifications of social innovation, e.g. depending on its nature (digital, technical, political, ethical, etc.), normative approaches and impacts (transformative, global, local), their focus of intervention (social innovation in medicine, health, education, urban transformation), product vs service or the process, etc. An attempt to systematise social innovations requires further study because of its wide scope and variety of forms. The existing division in the literature is not homogenous. The Atlas of Social Innovation (2018), the effect of cooperation between twenty-five partners, illustrates more than a thousand examples collected from all over the world, which also proves the topicality of the issue. On the basis of literature review (e.g. Benneworth & Cuhna, 2015; Adams & Hess, 2010; Mulgan, 2007; Andrew & Klein, 2017; Neumeier, 2012; Gerometta et al., 2005; Murray et al., 2010; Anderson et al., 2018) and the definitions proposed by organisations like, e.g. Bureau of European Policy Advisors; European Commission, OECD, Polish National Centre for Research & Development, the following definition of social innovations is proposed:

Social innovations are innovative solutions that have a predefined social objective, are used to meet specific social needs, lead to the development and strengthening of civic society, and are based on cross-sectoral and inter-area cooperation between actors, thereby also changing social relations.

**Fig. 1:**
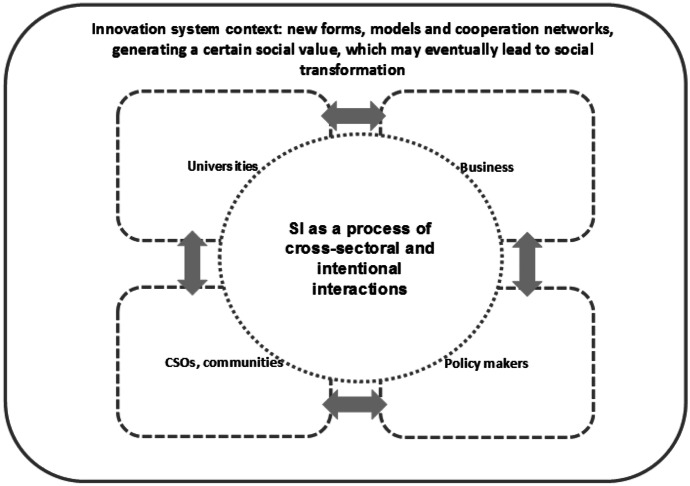
Social innovation as a process. Source: Author

Following this definition, I propose to consider social innovation in two ways: as a process and as an output of innovative activity. In the first case, we can say that social innovation is.

a total of intentional, responsible interactions of various interested parties, who share the common objective of finding a solution to a specific social problem (Fig. 1).

There appear new forms, models and cooperation networks, most often within a given area, generating a certain social value, which may eventually lead to social transformation. The idea of fair-trade, participatory budget, Living Lab concepts, a new care system for dependent persons, or home schooling can be examples of social innovation as a process.

**Fig. 2:**
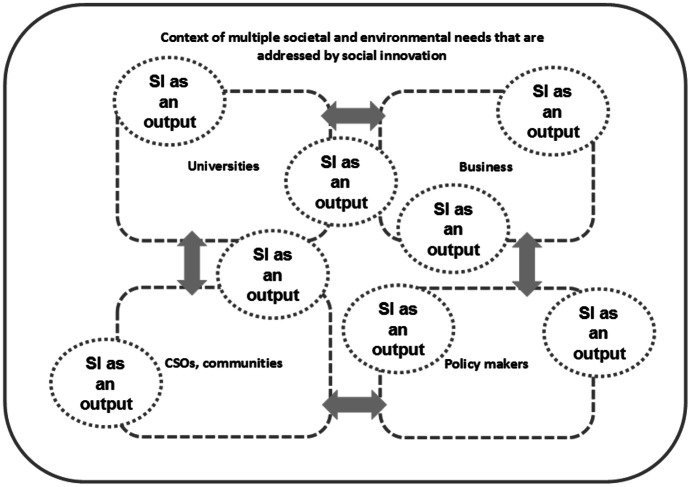
Social innovation as an output. Source: Author

In the case of the social innovation defined as an output, there are.

new or more effective solutions to a specific social problem, created within interdisciplinary and cross-sectoral cooperation, which are environmentally friendly and support social development (Fig. 2).

An example of this type of innovation will be certain products, services, tools which improve the quality of life, e.g. software for the blind and partially sighted, specific education programmes for excluded groups, e.g. children from rural areas, intelligent transport adapted for various passengers.

What the two approaches have in common is a social objective, which has not been achieved with the existing solutions, and the pursuit of a lasting change which will meet social needs and expectations. Social innovations are about to solve social problems, hence the importance of measuring their effectiveness. The existing rankings of innovativeness are primarily based on metric indicators and data, such as the number of patents, trademarks, outlays on R + D activities, number of scientific publications, etc. The effects of social innovation are mostly related to a systemic change which is not always clearly measurable. Likewise, it is difficult to assess the normative dimension of social innovation, e.g. the improvement of the quality of life. Therefore, measuring their effects is challenging and requires combining existing, measurable data with new aspects or factors of social innovation such as the creation of social value and social well-being. What is also an important issue is to determine the conditions enabling their development. Research on this topic focus on the identification of conditions and places in which social innovations emerge and explain how they can affect social change, what kind of tools may be applied by decision makers and key interested parties to ensure this development. The complexity of the problem of measuring the effects of social innovations results mainly from the fact that they are often hybrid in nature, complicated and emerge at the interface between various sectors and organisations. Moreover, social change occurs within a certain time perspective, therefore the products and results of a given project can be measured quite effortlessly, but its impact, i.e. the actual change, is not easily identifiable, and thus directly attributable to the effects of social innovations. The latter seems to be the hardest to investigate.

## New Approaches to Universities’ Missions and Their Role in Social Innovation

The new roles of universities in modern societies are the subject of both public and scientific debate. A number of new concepts have been developed throughout the last two decades. They reflect on the ongoing socioeconomic changes, including the modern concepts of innovation systems. They are also related to Mode 3 university that would ‘represent a type of organization that seeks creative ways of combining and integrating different principles of knowledge production and knowledge application (for example, Mode 1 and Mode 2), by this encouraging diversity and heterogeneity and by this also creating creative and innovative organizational contexts for research and innovation’ (Campbell et al., 2015, pp. 8). Just to list a few, e.g. entrepreneurial university (Clark, 2001), civic university (Goddard et al., 2016), developmental university (Arocena & Stutz, 2021) or the recent Fourth Generation University (Dewar, 2017). The increased attention is paid to different forms of universities engagement in societal problems through the third mission, commonly associated with such concepts and methodologies as community engagement, participatory or action research, service learning (and Science Shop), co-creation, citizens science, Living Labs, etc. (Table 1). Usually, they are discussed in contrast or in comparison to ‘traditional’ triple helix understanding of transferring knowledge and technology to the industry and society through commercialisation of knowledge. The notion of the third mission of university relates to the activity which contributes to the development of the economy and does not directly constitute the first or the second mission, which is education and scientific research. The precise definition of the third mission is problematic, because the same definition implies a different form of university activity, while, e.g. lifelong learning, open courses which are often called the third mission of university, are nothing more than educational activity. Likewise, the activities concerning commercialisation or technology transfer stem naturally from innovation activity which is based on scientific research. It can therefore be assumed that the third mission refers rather to a specific group of interested parties who are different from those engaged in the two traditional missions. Cavallini et al. (2016) define the third mission as relations (with the intention to exchange knowledge and productive interactions) with non-academic decision makers, and in particular with business, policymakers and society. It is a set of actions that allow for the production, use and application of knowledge and other university resources for the benefit of society, which are the integral part of the two basic university missions. Thus, it is the direct implementation of the assumptions of social responsibility.

**Table 1:** Selected types of engagement in social innovation (SI) throughout university missions

Missions	Methodologies and tools	Outcomes	Impact (internal and external)	Relations to QHM
Teaching and SI	SI pedagogy Educating about SI New education tools as SI: Life Long Learning MOOC	New curricula on SI SI integrated in curricula New digital tools implemented to education	Accommodating different educational needs and integrating vulnerable groups or with diverse backgrounds into university education Students as ‘change agents’, good citizens, advocating for SI Equipping society with knowledge relevant to deal with wicked problems	Delivering knowledge and skills related to SI to various stakeholders
Research and SI	Research on SI Research creating new SI	New theories and methodologies in SI Novel SI	New research networks and research partnerships Growing recognition of solution-oriented research Social impact of research included into the evaluation policy (e.g. REF in UK) Gap to be addressed due to lack of indicators for measuring impact of SI research	Establishing new channels for transdisciplinary research on SI SI included into regional strategies
Third mission and SI	Public/community/stakeholder engagement Community service Service learning Community-based participatory research (CBPR) Action-research, Citizen science, Science Shops (as methodology) Living Labs (as methodology) Promoting SI through Outreach and Science Communication Social entrepreneurship programmes	Novel SI Community/stakeholder engagement programmes and agendas Science Shop as university units Centres for Social Innovation established	New public–private partnerships for SI New research networks Knowledge democratisation Creating community of practice in SI Social impact of research included into the evaluation policy (e.g. REF in UK) Gap to be addressed due to lack of indicators for measuring impact of SI research	Mutual learning and the new channels of knowledge flow to and from the university allowing for new SI SI as part of regional innovation system New investments in physical and digital regional infrastructures on SI Regional leadership in SI
Fourth mission and SI*	Universities as open systems in relation to their environment and thus delivering social innovation Co-design, co-creation and co-delivery for sustainability SI as tool for implementing SDGs	Sustainability research and education programmes and curricula	All missions equally important and mutually enhancing New university rankings developed (e.g. Times Higher Education Impact Ranking) SDGs integrated within university strategy and all missions Accountability of universities ensured through appropriate governance and continuous exchange with policy makers, civil society, citizens, business Gap to be addressed due to lack of indicators for measuring impact of SI and impact of SI for sustainability	Opens system of education, research and innovation focused on the environment and sustainability Continues exchange between university and stakeholders Building regional capacity and resilience though SI

Source: Author

*Only novel approaches or new emphasis in comparison to third mission

Contemporary understanding of university engagement with communities is a complex phenomenon that is at the same time ‘a method (involving multiple partnerships and collaborative work), a principle (with mutual benefit at its core) and an objective (of contributing to societal development)’ (Farnell, 2020, p. 32). Universities are linked by numerous networks of dependencies and relations with different global, regional and local entities. When assessing the influence of universities on communities and society, we most often think of those situated in the immediate vicinity of university campuses. However, global impact has been possible thanks to technological progress. The analysis of key interested parties and the university environment is focused on the notions of relations, the natural environment, expectations and obligations.

A specific community becomes important for a university if both parties have expectations which can be met by mutual interactions or exchange. The meaning of the word ‘community’ here is closer to the definition of interested parties (stakeholders), i.e. groups or units which may influence or are under the impact of achievements within the implemented strategies. Therefore, the interested parties of a university are organisations, groups, or units which have a multitude of dimensions and features. Interested parties may thus be classified as internal and external, individual and collective, scientific and non-scientific. Farnell (2020, p. 32) also notices that the term ‘community’ is not necessarily limited to the local level: ‘Although it is easier to sustain productive relationships with partners that are geographical proximate, community engagement can also have regional, national and international dimensions’.

Admittedly, internal stakeholders are the nucleus of the functioning of a university, i.e. academics who should be independent of external influences. Students, as a key interested party, perform an important role in the contacts with the university environment, e.g. by internships, the popularisation of science, activities in scientific clubs. The administrative staff, in particular financial, personnel or technology transfer departments, are also increasingly professionalised, performing the function of a ‘guardian’ in the relations with the environment, and link the management staff with academia. Thanks to the potential of academic institutions it is possible to dynamize complex economic, social, cultural or technical processes. The social role of university may also include initiating new partnerships, establishing broad relations involving various types of organisations, communities, inhabitants, and thereby promoting citizenship. This is another field for the development of a higher education institution and for extending its role in shaping the future society. By developing cooperation with different stakeholders in a region and building networks of dependencies, it may create its image as a civic university.

Radinger-Peer (2019, p. 171) stress that in contrast to the first two pillars—research and teaching—regional engagement is a multifaceted and hard-to-demarcate phenomenon. The author argues that universities’ interactions with their respective region have a dual nature. ‘This duality refers, on the one hand, to contributions via linear, direct knowledge transfer activities (pipeline-dominated perspective), while, on the other, it entails contributions that emerge from formal and informal participation in regional networks, collective action and knowledge co-production with various actors from multiple backgrounds’.

Riviezzo et al. (2020, p. 31) propose a term *fourth mission* as ‘the promotion of social, cultural and economic development of the host community, that, in a very broad sense, leads to argue that university should contribute also to the quality of life perceived by the community itself’. The quality of life seems to be the essence of this new approach. Dentoni and Bitzer (2014) indicate for an increasing number of scholars that urge for a radically different understanding of the role of universities in societies, moving away from a sole focus on research and education tasks towards embracing what is referred to as a ‘fourth mission’ of universities. ‘In particular, universities are called upon to consider themselves as open systems in relation to their environment and actively seek the engagement of stakeholders, including municipalities, industry, civil society or development agencies’ Dentoni and Bitzer (2014, p. 3). The discussion of university engagement in social innovation is, therefore, shifting to the point where universities must inculcate social innovation in the teaching and research mission. They also need to conduct the necessary organisational transformations to invest in non-commercial social innovations which will benefit local communities (Bayuno et al., 2020, pp. 2). In this paper, I agree with this notion and focus on the need of universities to incorporate the societal and sustainability priorities in a systemic way and by this to play an active and leading role in QHM. Social innovation in university mission exists in literature, but it is still fragmented and related to particular case-studies approach or focusing on particular national or European initiatives or national programmes. The dominant model is still the lack of a strategy towards social innovation, short-term project-oriented approaches. Universities need new tools and model that will help to benefit from social innovation. As Benneworth and Cunha (2015) indicate that only when the university’s relationships with social innovators are closely aligned with broader strategic university interests, will the social innovation research process flourish.

Social innovation is usually oriented towards transformational changes; hence, the question issued by Klein (2017) ‘Can the participation of universities in the social innovation process contribute to the formation of a new society model?’ seems of key importance. On the one hand, the social mission of university should encourage them to seek and develop alternative solutions to crises and challenges. On the other, universities adopt strategies promoting an elite model of education and research, geared to competition. Thus, they consider their function and role in producing and disseminating knowledge within the existing institutional order, which does not lead to any real change in it. Despite that, examples of alternative activities can be shown. This is related to a new paradigm according to which what is recognised as crucial and necessary is the knowledge developed as part of co-construction, i.e. as a result of combining academic and practical knowledge generated by various stakeholders and innovation process participants. Therefore, universities may to a great extent contribute to the formation of more democratic and just society where the significance of knowledge is not determined by competitiveness and productivity but by the improvement in the quality of life and cooperation with citizens and local communities (Klein, 2017).

Anderson et al. (2018) emphasise that social innovation with the participation of universities account for a mere 14.9% projects covered by the Social Innovation Atlas, and research and education institutions made up only 21%. By contrast, non-government organisations took part in over 80% of social innovation, and private companies, or public institutions in 67%. The marginal involvement of research and education institutions raises the question of their role in the process of shaping and diffusing social innovation, especially as they are a pillar of innovation systems and a source of knowledge. Particularly untapped potential belongs to social sciences and humanities. As authors argue, HEIs seem to be ideal organisations for mitigating barriers to the implementation of social innovation. ‘Their role may include the following functions: intermediary in the institutional and political recognition of social innovation, as it often involves processes that change the existing pattern; research in the area of the evaluation of social innovation effectiveness and its scaling possibilities; an expert one, as well as advisory, mentoring, strengthening, supporting communities and lobbying for their benefit; logistic support, the available area, research equipment, incubation’ (Anderson et al., 2018, p. 51).

Similar approach is proposed by Benneworth and Cuhna (2015). They define university role in social innovation process as providing knowledge (existing or developed as part of the cooperation with the environment) to the creation of social innovation, sharing its tangible and intangible assets and advising social innovators and involving interested parties. Knowledge, support resources may be provided at various stages of creating social innovation and in different dimensions. The authors also argue that universities’ greatest contribution to social innovation comes when their relationships with social innovators, are closely aligned with wider strategic university interests. This however is still a great challenge. Benneworth et al. (2020) explore this failure by focusing on universities’ internal norms, value systems and ways of working, conceptualised as ‘institutional logics’ that frames how the organisation and its members interact with external actors.

In this paper, I agree with Grau (2017) that universities may fulfil a key role in social innovation with regard to solving (a) global problems, the reflection of which is the 17 Sustainable Development Goals of the global UN agenda, (b) local problems: social, cultural and economic, contributing to better development and competitiveness. In other words, this is about the activities which will lead to a socio-economic transformation and propose solutions related to the expectations of all social groups concerned. The participation of different stakeholders should be visible in particular at the formulation of new research agendas and the dissemination of their results (Benneworth, 2017).

Undoubtedly, strategic orientation towards social innovation within universities remains a challenge. It is important to design a strategic framework for action regarding various and often conflicting expectations of interested parties (stakeholders) while preserving autonomy and scientific excellence. The ongoing discussion on different levels is a clear sign that social innovations are a future issue.

## Research on the Potential of Polish Universities Regarding the Implementation of Social Innovations

### Methodology

The potential of an organisation is understood as the configuration of resources, abilities and attitudes, as well as the experience of an organisation used (or which can be used) for a specific aim. The elements indicated in the definition are practically all that an organisation has. These are static elements (resources and experience) and dynamic ones (abilities and attitudes). In the case of universities, we can say about tangible and intangible resources which, combined with the tradition and experience of cooperation with stakeholders in a region, may determine the activities of universities in the area of social innovation. Tangible assets include a university infrastructure (not only educational buildings, but greenery, libraries, laboratories as well) and also finances and an ICT base. Intangible resources comprise knowledge, the human capital of a university, its network of local, national or international links, presented values, etc. They are also visible in implemented research and educational projects, scientific publications or patent applications and growing companies. The university potential defined in this way mirrors its role in regional innovation systems in which knowledge is the most fundamental resource and the learning process is interactive, social in nature and created by many actors.

The research aimed to verify if the potential of Polish public universities was used to create and implement social innovation. My interest focused on social innovation addressed to internal stakeholders of a university (students and employees) but also to external ones. I wanted to define both the types of innovation developed and their scope and how they are embedded in QHM, what types of partnerships are created for social innovation. Moreover, the empirical part of the work serves to validate the main hypothesis hereof, which assumes that if Polish universities want to establish the effective system of cooperation with the social and business environment, they should build relevant structures and mechanisms supporting the development and implementation of social innovation in a region, thus contributing to the practice of university social responsibility. For the purpose of my research, university social responsibility is understood as the active presence at various levels of the country and civil society functioning, recognising the needs of all interested parties, shaping citizenship and democratic attitudes, protecting basic values important for an academic environment, integrating local society around the university. It means voluntary acceptance of social obligations by a university, which go beyond obligations arising from the applicable law. It also assumes greater openness to all internal and external interested parties as well as the natural environment in order to better shape the citizenship of students and to strengthen the impact of the university on the form and character of social development. This is a kind of foundation, attitude of academia towards the socio-economic environment, the manifestation of which are specific university activities constituting its third and fourth mission, i.e. that take into account the production, use and application of knowledge and other resources for the benefit of society. If social innovation is to be treated as a process, it will mean the new forms, models and cooperation networks with all relevant actors, and especially with the community within QHM. When we assume that social innovation results from traditional missions, it can take a form of concrete solutions transferred to the society within research or education activities. Social innovation can also develop within a university, implementing its social responsibility in relation to key stakeholders, i.e. students and university staff and take the forms of a new organisation and the evaluation of research (process), or social programmes for employees, e.g. on-site nurseries (product, result).This not a close list of possible dimensions as social innovation by a definition can develop in multiple ways.

The methodological framework is qualitative and interpretative. It seeks to gain insight into the experiences of Polish universities regarding the social innovation policies and practices. It also tries to explain the determinants of effective cooperation of universities with the regional stakeholders and was considered at several levels: university strategy, organisational culture, structure and management; key missions that is education and research and cooperation with external stakeholders.

The research conducted served also to implement several practical goals: to systematise knowledge on the implementation of social innovation at Polish universities, to identify socially relevant areas in which universities are involved, to pinpoint challenges facing universities in terms of building cooperation with the social and business environment and policy makers. The primary research tool was a survey questionnaire targeting the management staff of Polish universities. The scope of the conducted empirical studies has been limited to Poland and embraces all public universities, i.e. those supervised by the Minister of Science and Higher Education, and medical universities supervised by the Minister of Health. The questionnaire was addressed to 69 universities and 35 state higher vocational schools, i.e. a total of 104 HEIs, that is all public academic universities. The questionnaire referred to the activity of Polish universities in the period of 2012–2016. The desk study referred to the existing strategies, development plans and annual reports on university activities for 2016, 2017, 2018 and 2019. Sixty-three completed questionnaires were received in response (60%). Among universities which responded to the questionnaire were 16 state higher vocational public schools (UVOC), 14 classical universities (U), 14 technology universities (UT), 6 medical universities (UM), 5 universities in life science and/or agriculture (UL), 5 physical education academies (UPE), 3 university of business and economics (UE), and 1 pedagogical university (UPED). The questionnaire was sent by traditional mail and shared online by an open access tool, i.e. a Google Form.

The answers to the survey questions were mainly provided by the university staff employed in central organisational units, i.e. rector’s offices, representatives for the cooperation with the environment or for university social responsibility, centres of technology transfer and innovation, as well as marketing and promotion (outreach) centres. Research material corresponding to all thematic areas was collected on the basis of closed questions with a dedicated list of answers and open questions extending this list. When formulating the questions an attempt was made to limit their number and concentrate on the easily identifiable areas of university activities so to increase chances of getting enough answers that would make it possible to draw conclusions. The literature research carried out indicated a low level of understanding the idea of social innovation, which was important for the preparation of a relatively simple questionnaire and concentrating on the more recognised policies. The conclusions were developed on the basis of the questionnaire results as well as on the detailed desk research analysis that covered the available electronic material and university internet resources, including annual reports, development strategies, assumptions and results of competitions regarding social innovation. The procedure adopted and the results obtained allowed collecting coherent information on the level of interest in social innovation and the development of the third mission among Polish universities. The research outcomes constituted also the basis for the model of a socially engaged university.

### Social Innovation vs University Strategy, Organisational Structure and Management

Most universities (64%) indicated that the issue of social innovation is on the agenda of management bodies. The responders indicated diverse topics related to social innovation (Table 2), which, on one hand corresponds to the essence of social innovation that may relate to many areas of life. On the other hand, however, they are often so imprecise that they may suggest a low level of understanding the issue. Positive example can be illustrated by a following reply:

The issue related to the implementation of specific social projects by the university is repeatedly raised during sessions of the Senate or the Rector’s College. In their activities, the university’s authorities analyse and consider the need for social innovation both within the internal structures and outside the university. The subjects discussed most often concern: the introduction of changes in the organisation of services provided by the university, and also regarding new or improved methods of their rendering (new fields of study, opening of the university for the cooperation with the socio-economic environment, research and advisory services, new forms of social support, etc.).

**Table 2:** Summary of results of the research addressed to Polish public universities on their potential in social innovation (SI)

Respondents were 63 entities including: 16 state higher vocational public schools (UVOC), 14 classical universities (U), 14 technology universities (UT), 6 medical universities (UM), 5 universities in life science and/or agriculture (UL), 5 physical education academies (UPE), 3 university of business and economics (UE), and 1 pedagogical university (UPED)		
Level/dimension of investigation	Types of activities	Responses and/or statistics
Strategy, organisational culture, structure and management	Social innovation on the agenda of university management bodies	Yes for 64% of responders The responders indicated the following topics related to SI that are included into their agenda: Education programmes and curricula targeting various groups of recipients (Universities of the Third Age, Children Universities, Universities of Young Explorers, Intergenerational University, Academy of Seniors, open education, education for safety, local education, open lectures, academic classes, distance learning, lifelong learning) Measures to prepare students for the labour market (special curricula, dual studies, job fairs, entrepreneurship training) Measures for people with disabilities Programmes on ecology and environmental protection Projects in the area of health and promotion of a healthy lifestyle; culture (concerts, exhibitions) Internationalisation Measures for counteracting discrimination, reconciling work and family life, and regional development Participatory budget of a university
	Social innovation included in a strategy	Social innovation was included in the strategy of 61% of responders: 10 U, 13 UVOC, 3 UM, 8 UT, 3 UL, 2 UPE
	Expressed interest in implementation SI strategy provided that additional funding will be secured	Yes for 95% of responders: 14 U, 16 UVOC, 6 UM, 4 UL, 3 UE, 4 UPE, and 13 UT
	Programmes related to SI implemented	Yes for 78% of responders: 14 U, 13 UVOC, 6 UM, 3 UPE, 2 UE, 8 UT, 3 UL
	Monitoring social impact	Yes for 27% of responders: 6 U, 3 UVOC, 2 UM, 3 UE, 2 UT and 1 UL
	Internal programmes referring to SI e.g. on work-life-balance	Yes for 68% of responders: 9 U, 11 UVOC, 5 UM, 4 UL, 2 UE, 3 UPE, and 9 UT
Key missions: education and research	Curricula corresponding to the needs of economy, labour market and society	Yes for 93% of respondents. Universities claimed that they introduced new curricula corresponding to the needs of the economy, labour market and society
	Bachelor or master programmes with potential in cooperation with various entities	Yes for 82% of respondents. What is especially worth noting is a large number of positive responses of universities (12) which prefer cooperation with companies and a local government. State higher vocational schools have also indicated the cooperation with companies (12) and a local government (7), quite similarly in proportion to universities of life sciences, economics, physical education and technology
	Research related activities responding to societal challenges	Classical universities most often indicated the following research topics: progressing degradation of the natural environment (93%), health (92.8%), population aging (93%), youth unemployment (78%) and migration (43%). State higher vocational schools highlighted: health (50%), population ageing (31%), and the area concerning the natural environment (25%). All medical (100%) as well as physical education academies pointed to health and population aging. Interestingly, the area of health was also indicated by 7% of universities of technology. They most frequently selected degradation of the natural environment (78%) Among other areas pointed out by 17% of universities were: investment projects which would contribute to the practical education of students, research and analysis concerning cleanliness of cities, renewable energy sources, food safety, innovative use of waste products from the dairy industry, cyberspace, non-discrimination and social inclusion, efficient management of natural resources
	SI related to quality of life and sustainable development	Over 61% of universities participated in at least one project in partnership with public sector institutions of any level, an NGO or business, the objective of which was implementing solutions in the area of social innovation for the improvement of the quality of life. This kind of answer was provided by 10 U, 9 UVOC, 5 UM, 3 UPE, 3 UE, 5 UT, 2 UL 78% of respondents also confirmed their involvement in projects or initiatives aimed at responding to global challenges. In case of UT (11) and of UL (5), the area concerning sustainable development (e.g. reducing the level of resource use by making better use of available resources, extending product life or sharing) correlates with their specialisation, i.e. conducting applicable and implementation research. On the other hand, the implementation of projects aiming to improve the quality of life has not been indicated by this group of universities. This was a domain of UM (5) and UPE (4). The quality of life can therefore relate to human health and everyday functioning or general well-being and not to the possibility of using the latest technological solutions. As regards U (12) as well as UVOC (13), the responses are relatively balanced, which also reflects their specificity, i.e. the multitude of disciplines practiced and a strong attachment to the local environment respectively
Third/fourth mission understood as cooperation with external stakeholders	Cooperation with social economy enterprises	Yes for 43% of respondents that declared to ordering products/services in social enterprises or cooperated with them in a different form. These were 8 U, 12 UVOC, 3 UM, 1 UL, 3UT, 1 UE, and 4 UPE
	Conferences and seminars open to wider audience with the aim to share the knowledge	Yes for 89% of respondents
	The relevant actors for cooperation in SI	The vast majority, i.e. 89% of respondents indicated business institutions (e.g. companies and banks). The other group is other HEIs and entities of the R&D sector (87%). 57% of universities pointed to the cooperation with NGOs, whereas 11% mentioned other entities: hospitals, health centres, clusters, culture centres, business self-government, Ministry of Science and Higher Education, Ministry of Health and professional associations., boards of schools directors To be specific in term of cooperation with a third sector it was confirmed by 2 U, 10 UVOC, 4UM, 1 UE, 3 UL and 2 UT

Source: Author

Most universities (62%) indicated the presence of social innovation objectives in strategic documents, which does not, however, translate into the establishment or functioning of a unit or an individual/representative, whose activity would fit in with this are (Table 2). A mere 17% universities pointed out that the management of social innovation is within the competence of specific units (the Centre of Knowledge and Innovation Transfer, Social Responsibility Committee) or such positions as the rector’s representative for university social responsibility, vice-rector for science and development, or vice-rector for innovation. It was only 1 classical university, 3 state higher vocational schools, 2 universities of economics, 2 of life sciences and 1 physical education academy. Only 32 respondents were interested in the preparation of strategies for the development and implementation of social innovation provided the availability of funds.

The majority of universities (as much as 81%) admitted that they implemented projects in the area of social innovation in the years 2012–2016. Universities could indicate a maximum of three examples of such projects. More than 100 initiatives was collected, but the majority of them, in the author’s opinion, did not meet the requirements of social innovation assumptions but where rather related to what is typically named as third mission activities oriented on socially relevant areas like health, unemployment, environmental protection, social inclusion. This does not however indicate that social innovations are not implemented. It rather suggests that universities still have difficulties in defining them. It also relates to the following topic, that a large proportion of universities (75%) does not measure the social impact of science. These answers point to a significant gap in the activity of universities. It would be important to try to verify the long-term measures of universities in the area of social innovation in order to be able to adapt research and education activities more effectively to social needs.

With reference to university culture promoting socially relevant internal programmes (Table 2), 67% of respondents confirmed that they were introducing programmes and implementing procedures facilitating the reconciliation between family and professional life. When asked about incentives for employees promoting engaging in innovation, only 46% of respondents claimed that they do that mostly through internal regulations, dispositions or resolutions of college bodies, including e.g. financial (i.e. incentive payments, allowances, rector’s awards, medals, distinctions, internal grants), organisational (i.e. less obligatory teaching hours) and related to career promotion (i.e. extra points in the qualitative evaluation of employees). Only 30% of universities responded positively to each question related to implementation of social innovation as internal solutions.

### Social Innovation in Education and Research

Over 93% of universities declared that that they introduced new curricula corresponding to the needs of the economy, labour market and society (Table 2). Most universities (82%) carried out bachelor or master programmes in cooperation with various entities (Table 2), but especially with business and policy makers (local governing authorities). Merely 12 universities declared cooperation with NGOs. Universities have indicated variety of areas of their research that respond to societal challenges and admitted to supporting and implementing projects improving the quality of life and sustainable development. They can be clearly related to their research and education specialisation (Table 2).

The diversity of responses indicates a very broad activity of universities and a strong sense of mission in the creation of conditions supporting the quality of life at the same time. By far the largest number of initiatives are in the area of health, and these are not only programmes carried out by medical universities. In addition, universities have also pointed to projects concerning social exclusion, environmental protection, energy or urban space. Interestingly, most programmes declared as those raising the quality of life did not appear in the responses in the questionnaire as examples of social innovation. Only the area of adult and school-age education has been treated almost identically. Nearly 70% of universities responded that they carried out or participated in projects concerning sustainable development.

### Cooperation with External Stakeholders Relevant for Social Innovation

Polish universities cooperate with various stakeholders mostly in a traditionally understood ‘third mission’ approach. As many as 89% of universities also indicated that they organised meetings, conferences and debates for the social and business environment that tackled the socially relevant issues through open meetings about the economy, science festivals, scientific debates, entrepreneurship days, discussion panels, thematic conferences on health, education, the labour market, innovation development, local government congress, networking meetings, and participation in regional and local competitions.

All respondents pointed out local authorities (government and self-government) as a strategic actor for cooperation at the level of rectoral authorities. The vast majority, i.e. 89%, of universities indicated business institutions as well (e.g. companies and banks), and also other universities and entities of the R&D sector (87%). Fifty-seven percent of universities pointed to the cooperation with NGOs, whereas 11% mentioned other entities: hospitals, health centres, clusters, culture centres, business self-government, Ministry of Science and Higher Education, Ministry of Health and professional associations (Table 2).

Each type of university identifies strongly with a region or a city where it is located, which is proved by pointing to the local government as a key partner. This is probably supported by the tradition of cooperation and external conditions, e.g. existing bodies connecting university authorities with heads of cities or regions, the economic significance of a university for a region, cooperation within a regional innovation system. Business partners are as important for state higher vocational schools as local governments, which reflect the professional profile of universities. However, in the case of universities of technology, life sciences and economics, the nature of the studies conducted, including application ones, is important for the intensity of cooperation with selected partners. For classical universities, business is important primarily when it comes to offering internships and practical learning for students as well as running dual studies. The weaker cooperation with business can be observed at medical universities and physical education academies, which results from the specificity of education and research conducted. As in the case of local government authorities, cooperation with other R&D actors is also a natural form of developing innovative research. All types of universities mentioned third sector the least frequently. This is certainly an area that requires development. Universities were also asked about the most important problems of the city they were situated. The areas of active cooperation at local level are the following: spatial development, revitalisation; co-creating the city strategy, caring for the quality of life, e.g. clean air, fight against smog, the problem of aging society; health: activation, preventive healthcare; environmental protection; youth unemployment; public transport and transport; education; cultural integration; tourism; local development; migration; the outflow of the young to large agglomerations, negative demographic trends.

The analysis of selected national competitions referred to social innovation in 2013–2020, allowed drawing the following conclusions:

- A university’s participation in projects on social innovation is more often associated with the role of a partner rather than an initiator and coordinator of a project. This does not apply to competitions aimed at the university exclusively.
- Social innovation is primarily initiated by associations, NGOs or companies.
- The main areas of undertaken activities concern education and training available for all generations, including the popularisation of science, and also the health sector and the availability of selected services for people with disabilities or the excluded.
- Their outcomes are difficult to assess because not all programmes have been completed or the reports on the effects of social innovation and its real benefits have not been available.

### Model of a Socially Engaged University

**Fig. 3:**
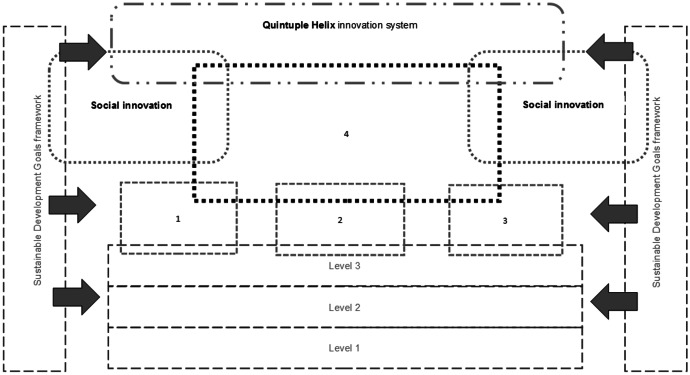
Socially engaged university model in quintuple helix innovation system. Level 1: university social responsibility included in a strategy, reflected in university missions and oriented both internally and externally. Level 2: university culture oriented towards dialogue, trust and supporting innovation and innovators (changemakers). Level 3: different structures and practices supporting social innovation. (1) Key missions: education, (2) key missions: research, (3) third mission: relations with external stakeholders, (4) fourth mission: co creation with stakeholders and communities for sustainability. Source: Author

**Fig. 4:**
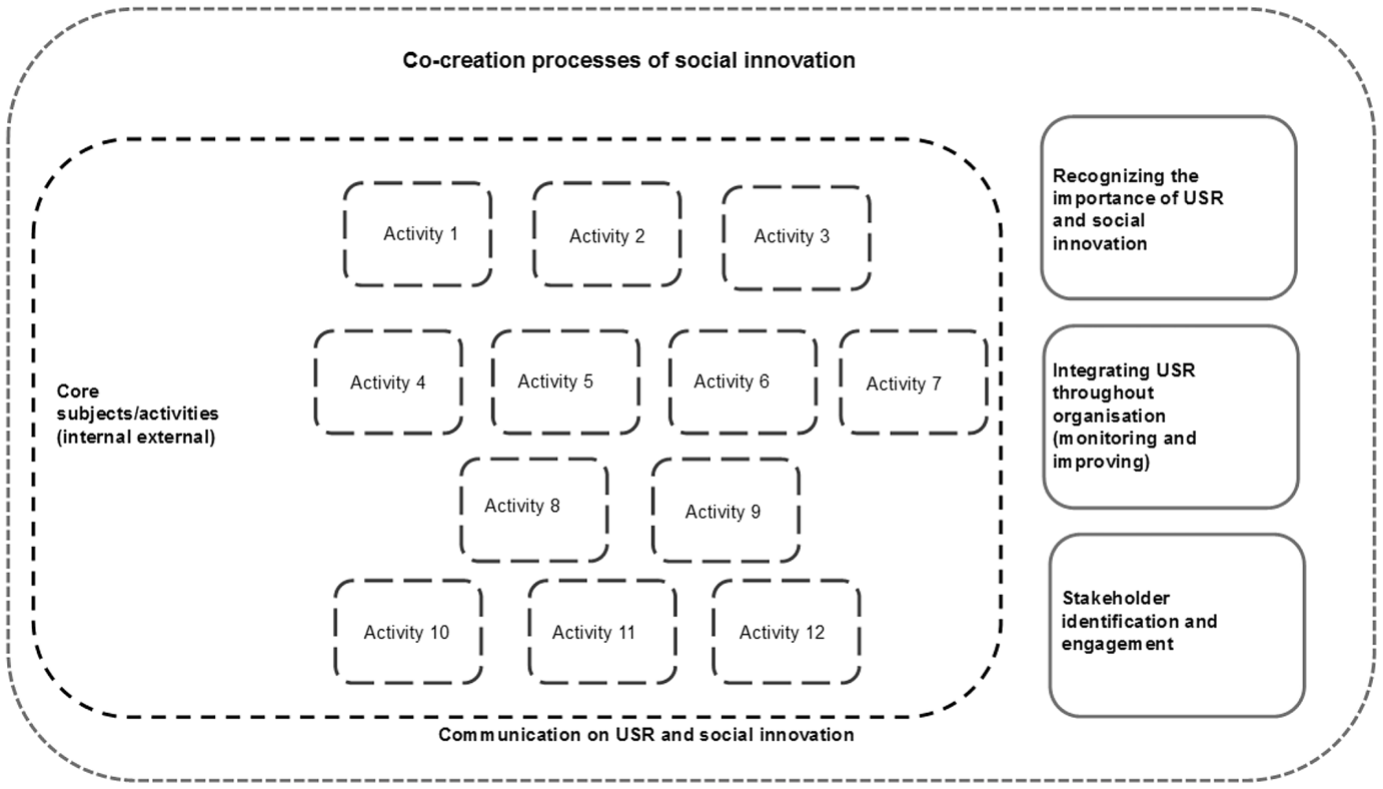
The overview of socially engaged university model. Activity 1: establishing a university-wide unit responsible for the development of social innovation in the university structure. Activity 2: integrating the UN Sustainable Development Goals with the strategy and mission of the university. Activity 3: supporting and giving priority to research of a high social impact. Activity 4: introducing the system of evaluation promoting and recognising prosocial activity of students and staff and their contribution to the development of social innovation. Activity 5: implementing equality and anti-discrimination policy. Activity 6: developing a social programme for university staff, particularly in terms of supporting employees caring for small children, dependent persons and the elderly. Activity 7: implementing equality and anti-discrimination policy. Activity 8: including students and PhD students in the development of university social responsibility. Activity 9: supporting the internationalisation of actions in the area of social innovation. Activity 10: supporting social entrepreneurship and socially innovative start-ups. Activity 11: sharing the university infrastructure with external organisations, particularly non-profit for the purposes of their statutory activities and provision of services. Activity 12: developing virtual communication tools, i.e. building an internet platform which will facilitate the process of establishing partnerships. Source: Author

Proposed in this paper model of socially engaged university (Figs. 3 and 4) does not refer to systemic solutions, or recommendations concerning appropriate legal regulation on a statutory level. This area requires further consultations, analyses, and research at the level of university governing bodies as well as science and higher education. A characteristic feature of the model is its orientation towards new kind of cooperation, co-creation and coproduction of knowledge within the so-called quadruple/quintuple helix. This process takes place within a regional innovation system, in the context of sustainable development principles, where the issues of environmental protection are a point of reference for the designed solutions.

It is a framework that can be designed and adapted accordingly to different regional, cultural and organisational contexts, as well as different disciplines and types of universities. It responds to the current understanding of the roles of universities in shaping and creating solutions responding to wicked problems of the twenty-first century.

The socially engaged model relates directly to the internal structure of a university and its organisational culture. It embraces, among other areas, human resource management, workplace safety, respect for the natural environment, equality and anti-discrimination policies and social impact of science evaluation. It is a practical tool of university social responsibility and as such it refers to the ways in which professors, scientists, administrative staff treat and interact with students, carry out research and resolve ethical dilemmas or resulting potential conflicts, and build relationships with the environment.

The effective cooperation between a university and the external environment in the process of social innovation is considered at the following levels: the university strategy; university structure; organisational culture; instruments and programmes related directly to its core missions; cooperation with the socioeconomic environment in the framework of the fourth mission understood as the co-creation with stakeholders and communities for sustainability.

The socially engaged university model assumes holding continuous dialogue with interested parties and citizens who should also participate actively in creating the programme of the development of social innovation and supporting the fourth mission. Starting point for this model implementation at a university is to identify stakeholders by their importance, influence, relations (formal and informal), origin (internal and external), connections with the university and ways of cooperation, as well as by different expectations and needs. One should not forget about relevant tools for communication that will facilitate the dialogue and reach the wider public.

#### The Core Assumptions of the Socially Engaged University Model

*Establishing a university-wide unit responsible for the development of social innovation in the university structure*, supervised by rector or the vice-rector of the university, and cooperating with the advisory body composed of the representatives of academics, administration, PhD students and students, as well as representatives of social and economic partners. Its tasks should include proposing an action plan, initiating and coordinating programmes, identifying good practices, drawing up relevant reports, monitoring and evaluating activities and recommending appropriate improvements and new solutions, collaborating with other centres home and abroad, co-creating the university strategy. The unit ought to be supportive in nature, because the development of social innovation should take place at the various levels of both the organisational structure and all basic missions. It is not obligatory for the model itself but the studies showed the low level of recognition of social innovation and such units would help to track progress in their development and monitor impact in a more sustainable way ( and not on ‘from project-to-project’ basis).

*Integrating the UN Sustainable Development Goals with the strategy and mission of the university*. Many examples of such strategies and challenges related to SDGs and universities are included in the GUNi Summary Report (2020). I propose to develop SDG’s related activities in six areas: research and education (new sustainable curricula, new research programmes oriented on green innovations etc.); resources efficiency management (water, energy, transport, CO_2_ emissions, energy effective buildings etc.); space related programmes (e.g. green and blue solutions, green infrastructure, environmentally friendly mobility on the campus and in the city); shaping the sustainability culture and promotion of health and wellbeing; coordination and sustainability management; and monitoring. For each of the are the superior aims, as well as relevant indicators should be developed.

*Introducing the system of evaluation promoting and recognising prosocial activity of students and staff and their contribution to the development of social innovation*. In the case of students, the system may give extra ECTS credit or micro-credentials for the implementation of projects involving social partners. With regard to researchers, such solutions are required at systemic level, but higher education institutions may, e.g. introduce financial awards or other distinctions, activate (also in collaboration with other actors) special grants and scholarships, even of symbolic value, which make it easier to implement projects of high social importance.

*Promoting organisational culture focused on cooperation, mutual learning, collegiality and the establishment of task forces*. This is one of the most complicated elements, because it refers to a great extent to the existing attitudes and values of a university, and those are extremely difficult to change. Its implementation is related to the current management and administration processes which should favour and promote higher-risk initiatives, ensure a relevant support system for researchers for the implementation of research and didactic projects, help introduce social innovation also at the internal university level. What has been recommended is the establishment of interdisciplinary teams that will jointly work on solutions using innovative tools and techniques, e.g. *design thinking*. Furthermore, it is necessary to provide a continuous training system and lead an internal information campaign on social innovations so that they become known and understood by the entire academic community.

*Developing a social innovation addressed to university staff*. The introduction of, e.g. a task-based work system, the organisation of on-site nurseries or preschools could be part of the programme. What are also important are financial incentives, e.g. preferential loans and credit, scholarships and subsistence allowances as well as other additional benefits. Certainly, the element of facilitating the reconciliation of professional and family life needs to be developed more intensely even for the sake of the aging population, which results in the increasing need to care for grandparents and parents of employees and other dependent persons.

*Implementing equality and anti-discrimination policy* in universities addressing gender equality, support for people with disabilities and religious, ethnical or sexual minorities such as Gender Equality Plans or Cultural Diversity Programmes.

*Supporting and giving priority to research of a high social impact*. What has been recommended in the proposed model in particular is the development of *citizen science or action research* with the active participation of external interested parties, especially non-profit organisations. They can be implemented within the so-called *Science Shops or Living Labs methodologies*.

*Including students and PhD students in the development of university social responsibility* through the development of curricula which offer students the opportunity to test in practice their competencies and knowledge and to acquire new skills through projects focused on the needs of a specific organisation or local communities (*service learning)*.

*Supporting the internationalisation of actions in the area of social innovation* by participating in international cooperation networks, initiating new project partnerships, taking part in international conferences and forums focused on public engagement. What has been recommended is the participation in, e.g. *The Living Knowledge Network, Global University Network for Innovation*, *European Universities Association, The Talloires Network, OECD Institutional Management of Higher Education Programme, Association of University Leaders for a Sustainable Future.* Universities should also intensify efforts to attract additional sources of funding to strengthen the international activity of students.

*Supporting social entrepreneurship and socially innovative start-ups.* A university may develop this form of social engagement by, e.g. activating programmes of courses or training dedicated for students, PhD students and researchers in cooperation with institutions supporting knowledge transfer and entrepreneurship, for instance scientific and technological parks. What has also been proposed is the organisation of competitions on incubating social innovation.

*Sharing the university infrastructure with external organisations, particularly non-profit for the purposes of their statutory activities and provision of services.* This requires the implementation of relevant regulations, including the establishment of safety and liability rules, as well as preferential fees. An innovative idea could be also designing a common space at the university, a project centre which will be a place to meet and implement joint projects of the university and selected organisations. It ought to be situated in such location that will guarantee an easy access. In the case of services, university staff may become consultants of selected organisations, share expertise and knowledge, promote social innovation and explain its mechanisms, consult new ideas, support as mentors, brokers and facilitators selected organisations which implement innovative solutions.

*Developing virtual communication tools, i.e. building an internet platform which will facilitate the process of establishing partnerships.* Cooperation with the environment takes place mostly through interpersonal relations, more rarely on a systemic basis. The structure of university is often complex, and the number of units responsible for various areas of activities makes it difficult to reach individuals with specific authorities or interested in particular subjects. Thus, the proposed tool should facilitate contact between various interested parties on a ‘one e-mail address, one phone’ basis. One of the first stages of building a dialogue platform is identifying groups (researchers, administration employees) at the university, who wish to create their profile and determine fields and conditions of cooperation with selected stakeholders. The base should be continuously expanded, but on a voluntary basis and in line with the actual need to lead specific initiatives. Simultaneously, the platform should also enable external interested parties to post their profiles and specific research questions, needs, cooperation offers in order to maintain a two-way flow of information. Implemented projects may be found as examples of good practices and at the same time may serve as an element of promotion of the university’s activity.

The implementation of the model involves the need to build a sustainable vision of development related to specific objectives to be achieved and it requires to adopt the following steps adapted from Pukka (2017, p. 74). It should be preceded by the assessment of various types of needs of both the university and the environment, combined with an evaluation of the resulting opportunities, possibilities as well as the identification of strengths and weaknesses. What is necessary is the identification of good practices in different dimensions, because the university output in terms of cooperation with the environment is usually very broad. The activity map confronted with the needs may show potential gaps which can be addressed and those will translate into the definition of objectives and target groups for the activities undertaken. The next stage is to determine a strategy, a road map and an action plan, and to match them with the needs at the level of the structure and internal organisation of the university, as well as the tools needed for contacts with the environment. The implementation stage should be combined with monitoring and evaluation which can be carried out in various fields. The first element of this process ought to be to determine the recipient of the evaluation.

## Summary of Research Results

The implementation and/or co-creation of social innovation by Polish universities is an area that is not sufficiently recognised due to the lack of a clear definition of social innovation and the methodological challenges related. It reflects the ongoing debate regarding the actual meaning of this phenomenon and lack of consensus on its definition (Benneworth & Cuhna, 2015; Adams & Hess, 2010; Mulgan, 2007; Mihci, 2019; Murray et al., 2010; Anderson et al., 2018). As already stated, social innovations are a qualitatively new element in the process of socio-economic evolutionary development based on knowledge and innovation. Social innovation is rarely mentioned in the strategy of a university. In practice, there are no units responsible for social innovation or support instruments (financial, organisational, motivating) which would relate to it directly. The conclusions drawn from the conducted research are not unequivocal due to a relatively poor theoretical and source material covering this sphere of Polish universities activity. The creation of social innovation takes place in cooperation with other actors, which is often associated with the third university mission. On the other hand, the social dimension of innovation relates also to the issue of university/science social responsibility, i.e. such activities that respond to the needs of selected interested parties and enhance the impact of universities on the shape and nature of social development. It should be emphasised that these activities can occur at the level of the all university’s missions. As Riviezzo et al. (2020) argue, there is a need to assess holistically and systematically the impact of different universities’ missions and activities on the society and their contribution to sustainability. Dentoni and Bitzer (2014) also propose to move away from a sole focus on research and education tasks towards embracing what is referred to as a ‘fourth mission’ of universities. Those findings proof to be relevant for Polish universities as well.

The analysis of the answers to the survey questions provided by universities as well as the desk research allows a general assessment of creating and implementing social innovation. It should be pointed out that not all socially innovative activities run by HEIs are reflected in available source materials. In other words, it is very likely that social innovations are created and implemented by universities, but the information about them may only be available in internal reports or statements, including those concerning the implementation of selected projects submitted to the institutions that finance them. The insufficient promotion of social innovation may result from the lack of a coherent information flow system as well. The universities investigated, in most cases, are large institutions which often carry out several hundreds of projects concurrently, hundreds of activities within the category of research and training as well as cooperation with the environment. In addition, innovative activities are not sufficiently promoted; there are no information sets and data repositories in this field. Another reason is the lack of a coherent policy in this regard or an action plan at most Polish universities. Social innovation activities at Polish universities happen to be scattered among various structures. This area is often assigned to the rector, vice-rector or representative, which is connected to the decision-making process rather than a systemic approach to the issue of social innovation development.

All universities emphasise the importance of cooperation with the environment and are aware of their role in the domestic, regional or local economy. They also point to other aspects of their usefulness for a given area, including social ones and those of opinion-formers, the development of human resources for the economy, support and promotion of culture, environmental protection, public health protection, cooperation with employers, development of the urban space, education growth, etc. Regional engagement is in fact a multifaceted phenomenon and is characterised by its dual nature referring to direct knowledge transfer as well as formal and informal participation in different regional networks, collective action and knowledge production processes (Radinger-Peer, 2019). The scope of the applied activities is diverse and certainly depends on the specialisation of a university and its conducted research. It is therefore difficult to make a direct comparison between medical universities and technology-oriented ones or comprehensive/classical universities and those of human sciences and economics. State higher vocational schools, located in smaller urban centres have different specificity and their role is definitely local or regional and less often national. In contrast to large universities, their cooperation with social partners is frequently more visible, because they operate in smaller communities and they are usually the only state university in a given *poviat* (county), and also the place where local elites are concentrated. Nevertheless, it can be pointed out, with regard to public academic institutions, that there are no systemic measures, i.e. none of the investigated universities has a strategic and comprehensive programme of cooperation with the environment, including in the field of shaping social innovation. Moreover, what usually dominates is a cooperation model of a knowledge transfer type and not a co-creation one, i.e. universities promote activities consisting in providing knowledge or sharing other resources, carrying out educational projects addressed to various age groups and popularising science. Meanwhile the current model puts considerable emphasis on the active cooperation and participation of different actors, each of which has a specific type of knowledge and powers, and new solutions are produced in the co-creation process and not by a linear model of knowledge transfer from the university to the environment (Arnkil et al., 2010; Cavallini et al., 2016; Trencher et al., 2013). The third mission is understood at Polish universities as the establishment of cooperation in which the university plays a dominant role and provides solutions, needed skills or competences, or promotes science education among the general public. Therefore, universities support the development of such units as technology transfer centres, careers offices, academic incubators, Colour Universities, Universities of the Third Age and science festivals; they organise open lectures, charity events, or promote student voluntary service. They do not always directly translate into the creation of social innovation.

It should be highlighted that universities are aware of their social role expressed by cooperation with the environment and strive to implement social responsibility at internal university level. This is achieved through social programmes for students or staff, including the ones facilitating the reconciliation between professional and family life, on-site nurseries or preschools, children’s play areas, free sports passes, clear policies for professional promotion, support for the development of competences and qualifications, etc. In November 2017, on the initiative of the Ministry of Development in cooperation with the Ministry of Science and Higher Education, (merely) 23 universities signed the Declaration of University Social Responsibility, which in late 2020 has had over eighty signatories. However, the effects of the actions of this network are not yet recognisable. Social innovation is still rarely the subject of scientific debate.

While referring to the use of potential for the development of social innovation by universities, it should be emphasised that it is mostly local. This is evidenced by the multitude of cooperation forms between university and partners and the wide range of topics of the indicated research and education initiatives. Scientific specialisation is certainly important for the development of these relations—their intensity is greater for the area of health, life sciences, materials sciences, ICT, etc. All the HEIs that participated in the research play a significant role in shaping socio-economic development, regardless of the size of the university, although this can surely affect the scope and extent of activities. The universities like state higher vocational schools focus often on local challenges, including on the labour market, and universities radiate not only to the region but also to the country. However, it should be emphasised the global fulfilment of the universities’ social mission can be achieved through addressing the Sustainable Developments Goals also on a local level (Grau et al., 2017) The dualism of activities results from contemporary expectations and development trends. The urgency of the sustainability issues is reflected in quintuple helix model (Carayannis et al., 2019) and sets the stage for sustainability priorities and considerations for knowledge production and innovation.

Social innovation created by universities in relation to the proposed definition (that is, *innovative solutions that have a predefined social objective, are used to meet specific social needs, lead to the development and strengthening of civic society, and are based on cross-sectoral and inter-area cooperation between actors, thereby also changing social relations*) can be therefore divided into social innovation inside the academia, social innovation developing in cooperation with stakeholders and social innovation which is the result of the research conducted by scientific units and transferred to the environment. The initiatives indicated (Table 2) are certainly important in the area of the development of social innovation at Polish universities, but what should be clearly shown is the existing gap in universities’ strategic measures and the need to build comprehensive programmes and models of cooperation in line with Western universities. The analysis of the results of the implemented projects regarding social innovation has been proposed as the next phase of research. A proper time perspective will make the further analysis easier. The reform of higher education that is being implemented under the name University 2.0 will be certainly of significant importance. It offers a series of substantial changes in the functioning of universities and the evaluation of scientific activity and may reformulate the organisational culture of universities and the assessment of education and the research conducted. Contemporary socio-economic challenges need non-standard and innovative solutions, which requires that a university should not only develop and be active in social innovation, but also an overall conception corresponding to the current approach to university social responsibility. *Universities that will build effective and lasting relationships with the socio-economic environment may become leaders in the development of social innovations which affect favourably the quality of life in a region. Therefore, there is a reasonable need to propose a model of a socially engaged university, involved in relations with the environment in the context of the quadruple/quintuple helix model*. This involvement through social innovation in social and economic development put more emphasis on concentrating social well-being and contributing to a new society model and it demands a transformation in existing institutional order (Klein, 2017). For incubating and supporting social innovation at university, what is needed is a system of coordinated activities facilitating cooperation with the regional environment which will lead to the increased involvement of a university in the implementation of social innovation. Direct involvement of researchers in relations with the inhabitants of a given region or city allows for better understanding of social expectations, doubts and priorities. What has been recognised is that the impact of research on social, economic and cultural development should be maximised wherever possible. Hence, the importance of the development of participatory research and new forms of education involving students in cooperation with social partners. This can be achieved through aligning such activities with wider strategic university interests (Benneworth & Cuhna, 2015). Undoubtedly, strategic orientation toward social innovation within universities remains a challenge for Polish universities.

## Discussion and Conclusions

This article assumes that universities should play an active role in the implementation of social innovation, in line with the existing new paradigm. It recognises unofficial, uncodified knowledge, belonging to various organisations and communities, as equally relevant in the process of creating innovation. In this new paradigm, the knowledge development is a result of combining an academic and practical knowledge generated by various stakeholders and the significance of knowledge is not determined by competitiveness and productivity but by the improvement in the quality of life and cooperation with citizens and local communities (Klein, 2017). In other words, universities should co-create solutions that meet different social needs. The literature examination aimed to show contemporary mechanisms of creating innovation by taking account of their network and bottom-up nature, which is departing from the conception of the triad universities-business-state to the model of the quadruple/quintuple helix universities-business-state-civil society institutions, in which an active participation of citizens is becoming increasingly important (Arnkil et al., 2010; Carayannis & Campbell, 2012; Carayannis & Grigoroudis, 2016; Carayannis & Rakhmatullin, 2014). Today’s global challenges have been shown to need interdisciplinary and unconventional solutions, the bases of which are actual needs of specific communities. ‘Tailor-made’ solutions with the active participation of interested parties are a significant feature of social innovation. They allow to form a more inclusive, democratic innovation system based on a dialogue and reflecting also values of the society. They also emphasise the importance of knowledge in creation of social well-being and in general the quality of life. Cross-sector or multi-actor collaboration could significantly contribute to a local or regional transition to sustainability (Trencher et al., 2013; Cavallini et al., 2016).

The source literature shows that new forms of innovation require universities to build effective structures of cooperation with the environment, and also to develop new forms of research and curricula which will be responsible for local, regional and global challenges, including those of the natural environment (Anderson et al., 2018; Baran, 2016; Benneworth & Cuhna, 2015; Benneworth et al., 2020; Dentoni & Bitzer, 2014; Goddard et al., 2016).

Main challenges for universities to participate in the progress of social innovation development are the following:

- Proper definition and understanding of the essence of social innovation, which is important given the diversity of definitions and the still low participation of science in their generation
- Incorporation of social innovation to the university mission and strategy, research agendas and curricula, and also to the activities constituting the third university mission
- Departure from ad hoc activities in favour of regular and systemic support for social innovation development
- Departure from planning top-down measures, which often engage local communities, but are designed for the needs of universities, not their partners, in favour of bottom-up design with the active participation of social organisations treated as equal partners

The support of social innovation by universities means that they are open, friendly and creative. Social innovation should become a stimulus of sustainable social, cultural, economic, political and environmental development. The formation of a fair and equal society through innovation is also about being a socially responsible employer. In this case, the internal potential is not fully exploited, because the highly hierarchical and bureaucratic structure is a significant barrier for social innovators (Baran, 2016).

Assuming that today’s innovation processes are mostly based on the growth of soft factors, including knowledge and its use in direct interactions, the development of a system for managing relations with university stakeholders seems to be key also for the social innovation process. Having resources is not sufficient; they have to be an active factor supporting the development of a given territory. The universities are therefore encourage to fully embrace the societal impact agenda, to engage with others across the broad spectrum of the research ecosystem; to develop open, explicit and transparent reward systems that include the value of all kinds of impact and to ‘continuously seek to support and promote societal impact as a dynamic, open and networked process in a culture of sustained engagement and co-production of knowledge’ (Spaapen & van der Akker, 2017). A dynamic environment in which universities play a significant role becomes the source of innovation, but what should be taken care of is cooperation between all four helices, i.e. government, business, authorities and society. The social role of university may be establishing far-reaching relations encompassing various types of organisations, communities and inhabitants. By ensuring cooperation with various interested parties in a region and building the network of dependencies, a university may create its image as a civic academy (Goddard et al., 2016; Nowakowska, 2011; Reichert, 2006).

This paper attempts to address the gap of relatively few studies on institutional change and incentive structures that influences the ability of universities to engage in social innovation and the gap of identifying connection between social innovation concept and the QHM framework.

The current European Universities Association’s vision of future universities states that:

‘Europe’s universities will make human-centred innovation their trademark, aiming to achieve sustainability through cooperative models. They will engage in co-creation of solutions with a wide range of partners and with the purpose of meeting common challenges and making a demonstrable difference to society through technological as well as social innovation. As such, universities will play a leading role in innovation ecosystems. They will bring together stakeholders around a common vision, bridging different cultures spanning from academia, business and start-ups, to civil society and the social and cultural scene. They will also reinforce their contribution to the development of knowledge and skills together with partners in the ecosystem’ (EUA, 2021, p. 9).

When analysing the solutions adopted in other countries (e.g. National Coordinating Centre for Public Engagement in the UK, Campus Engage in Ireland), one should emphasise that the regulatory system of higher education in conjunction with public policies is often crucial for supporting the development of social responsibility. The amount of outlays on science, the financing system of research, the tradition of cooperation between universities and businesses appear to be obvious factors.

In the case of social innovation and activities concerning social engagement, the determination of the impact of the university on regional development, social well-being and ultimately the generation of social change requires further research and development of the tools. ‘While their future cannot be planned, the tools they have at their disposal to meet the future can be improved. In order to thrive, universities need the right framework conditions: academic freedom, institutional autonomy, sufficient and sustainable funding and efficient support for collaboration’ (EUA, 2021, p. 10). It is therefore proposed by the author, wherever possible, to select quantitative indicators in a classical, project approach, i.e. embracing product indicators (adopting tangible, numerical, monetary units), result indicators (related to direct and immediate effects of programmes for HEIs and potentially also for interested parties, providing information on changes, e.g. in behaviour, potential and activity) and those of impact (qualitative) adopting short- and long-term perspectives. It has been assumed that product and result indicators should be monitored once every six months or once a year, whereas impact indicators ought to be verified in a several-year perspective, e.g. with regard to the term of office of the university authorities. The catalogue of activities and indicators should not be closed as it has to be deeply related to the actual university activity and its specificity. Therefore, in order to investigate social innovativeness, what should be applied are both ‘product’ indicators (kinds and types of research projects or training programmes) and those concerning social relationships with the environment, related to the formation of civil society and responsibility for inhabitants and their quality of life.

Based on the above considerations and with respect to the dynamic development of theory and practice in the field of social innovation, suggestions for further research can be offered. One is the development of social innovation in relation to QHM with focus on different roles of stakeholders at different stage of process of social innovation. The next could be defining tools and methodologies for measuring the effectiveness of the social innovations. This implies another challenge, i.e. the proposition of the metrics and indicators capturing not only their usefulness but also regarding an actual change (in various contexts, e.g. legislative, organisational, procedural, mental). A research problem which could be useful particularly for policymakers in a region would be to examine the relationship between the innovation level in the region and the functioning of the quadruple/quintuple helix model and to identify the correlation between selected indicators. It would be significant to show whether the intensity and quality of the network of connections between the helices (different stakeholders in the region) affect innovation indicators and how they change depending on the quality and importance of selected helices. A recommended research aspect will be also a detailed analysis of the collaboration level between universities and other actors of the innovation process, with particular attention to civil society organisations.

In 1992, over 1700 scientists affiliated to the *Union of Concerned Scientists* appealed to humanity to express their concern about the fate of the world. Twenty-five years later, the article which was published in the journal *BioScience* was signed by 15 thousand representatives of the scientific world from 184 countries. In an open letter addressed to humanity, its authors warn against the negative consequences of people’s actions that can be fatal to all human beings (Ripple et al., 2017). Scientists call on all people, and also on leaders to take action to reverse these negative trends. Among the proposed solutions were the establishment of more nature reserves, the adoption of legislation to facilitate nature protection, running educational programmes or an increased use of renewable energy sources. The appeal concluded with a call for cooperation to create transparent and practical solutions and respect for diversity, which will contribute to greater social justice. These words are in line with the idea of social innovation that seems to be the solution humanity needs today.

## Footnotes

1 This statement was formulated during the Social Innovation Summit 2014 in San Francisco.

## References

[CR1] Andrew, C., & Klein, J. L. (2010). SI: What is it and why is it important to understand it better. ET10003. *Ontario Ministry of Research and Innovation*. Toronto

[CR2] Alfonso, O., Monteneiro, S., & Thomson, M. (2012). A growth model for the quadruple helix. *Journal of Business Economics and Management,**13*(5), 849–865.

[CR3] Anderson, M. M., Domanski, D., & Howaldt, J. (2018). Social innovation as a chance and a challenge for higher education institutions. In J. Howaldt, Ch. Kaletka, A. Schröder, & M. Zirngiebl (Eds.), *Atlas of Social Innovation – New Practices for a Better Future* (p. 50–53). Sozialforschungsstelle.

[CR4] Arnkil, R., Järvensivu, A., Koski, P., Piirainen, T. (2010). Exploring quadruple helix. Outlining user-oriented innovation models. Final Report on Quadruple Helix Research for the CLIQ project Co-financed by European Regional Development Fund Made possible by the INTERREG IVC Programme. Report available at ResearchGate. https://www.researchgate.net/publication/265065297_Exploring_the_Quadruple_Helix. Accessed 15 January 2021.

[CR5] Arocena, R., Sutz, J. (2021). Universities and social innovation for global sustainable development as seen from the south. *Technological Forecasting & Social Change 162*, 120399. 10.1016/j.techfore.2020.120399 PMC7554489 33071365

[CR6] Baran, M.(Ed.) (2016). Building the capacity of social innovation in higher education. file:///C:/Users/joann/Downloads/SOCIAL_INNOVATION_IN-_HIGHER-_EDUCATION_IN_CENTRAL_EUROPE_REPORT_FULL-1.pdf

[CR7] Bayuno, B. B., Chaminade, C., Göransson, B. (2020). Unpacking the role of universities in the emergence, development and impact of social innovations – A systematic review of the literature. *Technological Forecasting and Social Change 155*, 120030.

[CR8] Bellandi, M., Donati, L., Cataneo, A. (2021). Social innovation governance and the role of universities: Cases of quadruple helix partnerships in Italy. *Technological Forecasting & Social Change 164*, 120518.

[CR9] Benneworth, P. (2017). The role of research in shaping local and global engagement. In Towards a Socially Responsible Higher Education Institution: Balancing the Global with the Local,* GUNI Report* (p. 249–259). Girona.

[CR10] Benneworth, P., Cunha, J. (2015). Universities’ contributions to social innovation: reflections in theory & practice. *European Journal of Innovation Management*, Vol. 18 Iss 4, 508–527

[CR11] Benneworth P., Cunha J., Cinar R. (2020). Between good intentions and enthusiastic professors: the missing middle of university social innovation structures in the Quadruple Helix. In Farinha, L., Santos, D., Ferreira, J. J., Ranga, M. (Eds.), *Regional Helix Ecosystems and Sustainable Growth. The Interaction of Innovation, Entrepreneurship and Technology Transfer* (p. 31–44), Springer.

[CR12] Boelman, V., Kwan, A., Lauritzen, J. R. K., Millard, J., & Schon, R. (Eds.). (2014). Growing Social Innovation: A Guide for Policy Makers, a deliverable of the project: The theoretical, empirical and policy foundations for building social innovation in Europe (TEPSIE), European Commission – 7th Framework Programme, Brussels: European Commission, DG Research. The Young Foundation. Available at https://youngfoundation.org/wp-content/uploads/2015/04/YOFJ2786_Growing_Social_Innovation_16.01.15_WEB.pdf. Accessed 12 October 2017.

[CR13] Carayannis, E. G., & Campbell, D. F. J. (2012). Mode 3 knowledge and Quadruple Helix: Toward a 21st-fractal innovation ecosystem. *International Journal Technology Management,**46*, 201–234.

[CR14] Chesbrough, H. (2003). *Open innovation: The new imperative for creating and profiting from technology*. HBR School Press.

[CR15] Campbell, D. F. J., Carayannis, E. G., & Rehman, S. S. (2015). quadruple helix structures of quality of democracy in innovation systems: The USA, OECD countries, and EU member countries in global comparison. *Journal of Knowledge Economy*. 10.1007/s13132-015-0246-7

[CR16] Carayannis, E., Grigoroudis, E. (2016). Quadruple innovation helix and smart specialization: Knowledge production and national competitiveness. *Foresight and STI Governance*, 10.17323/1995-459x.2016.1.31.42

[CR17] Carayannis, E. G., & Rakhmatullin, R. (2014). The quadruple/quintuple innovation helixes and smart specialisation strategies for sustainable and inclusive growth in Europe and beyond. *Journal of the Knowledge Economy,**4*, 221–239.

[CR18] Carayannis, E. G., Acikdilli, G., & Ziemnowicz, C. (2019). Creative destruction in international trade. Insights from qudruple and quintuple innovation helix models. *Journal of Knowledge Economy*, Vol. 11, p. 1489–1508 (2020). 10.1007/s13132-019-00599-z

[CR19] Carayannis E. G., Dezi L., Greogri G., Calo E. (2021). Smart environments and techno‑centric and human‑centric innovations for Industry and Society 5.0: A quintuple helix innovation system view towards smart, sustainable, and inclusive solutions. *Journal of the Knowledge Economy*. 10.1007/s13132-021-00763-4 10.1007/s13132-021-00763-4 PMC7903376 40477337

[CR20] Carayannis E. G., Draper J., Bhaneja B. (2020). Towards fusion energy in the Industry 5.0 and Society 5.0 context: Call for a global commission for urgent action on fusion energy. *Journal of the Knowledge Economy*. 10.1007/s13132-020-00695-5 10.1007/s13132-020-00695-5 PMC7529587 40477087

[CR21] Cavallini, S., Soldi, R., Friedl, J., Volpe, M. (2016). Using the quadruple helix approach to accelerate the transfer of research and innovation results to regional growth, Committee of the Regions, European Union. Online report. Available at https://cor.europa.eu/en/engage/studies/Documents/quadruple-helix.pdf. Accessed 13 March 2019.

[CR22] Clark, B. (2001). The entrepreneurial university: New foundations for collegiality, autonomy, and achievement. *Journal of the Programme on Institutional Management in Higher Education, Higher Education Management, Education and Skills, OECD, 13*, 2, 9–25. http://www.oecd.org/education/imhe/37446098.pdf

[CR23] Dentoni, D., & Bitzer, V. (2014). The role(s) of universities in dealing with global wicked problems through multi-stakeholder initiatives. *Journal of Cleaner Production,**106*, 68–78. 10.1016/j.jclepro.2014.09.050

[CR24] Dewar, J. (2017). University 4.0: Redefining the role of universities in the modern era, *Higher Education Review, Magazine*, Available at https://www.thehighereducationreview.com/magazine/. Accessed 19 April 2019.

[CR25] European Commission, (2017a). Communication on a renewed EU Agenda for Higher Education. Brussels: Commission of the European Communities, COM (2017) 247. Assessed 15 January from: https://ec.europa.eu/education/sites/education/files/he-com-2017-247_en.pdf

[CR26] Edquist, C., Luukkonen, T., & Sotarauta, M. (2009). Broad-based innovation policy. In Taloustieto Oy (ed.), *effectiveensstion of the Finnish national innovation system – full report*, p. 11–69, Helsinki, www.evaluation.fi. Assessed 15 December 2018.

[CR27] Etzkovitz, H., Leydesdorff, L. (Eds.) (1997). *Universities and the global knowledge economy. A. Triple helix of university – industry – government relations*. Pinter, London and Washington.

[CR28] Farnell, T. (2020). *Community engagement in higher education: Trends, practices and policies*. Publications Office of the European Union. 10.2766/071482

[CR29] Florida, R. (2004). *The flight of the creative class, The New Global Competition for Talent*. Harper Business.

[CR30] Foray, D. (2014). From smart specialisation to smart specialisation policy. *European Journal of Innovation Management,**17*(4), 492–507.

[CR31] Fostering Innovation to address social challenges, workshop proceedings, OECD (2011). http://www.oecd.org/sti/inno/47861327.pdf. Accessed 30 January 2021.

[CR32] Gaczek, W. (2009). *Gospodarka oparta na wiedzy w regionach europejskich*, Polska Akademia Nauk Komitet Przestrzennego Zagospodarowania Kraju, Warszawa.

[CR33] Gerometta, J., Haussermann, H., Longo G. (2005). Social Innovation and Civil Society in Urban Governance: Strategies for an Inclusive City’, *Urban Studies*, vol. 42:11, 2007–2021

[CR34] Goddard J., Hazelkorn, H., Kempton, L., Vallance, P. (2016). The Civic University: The Policy and Management Challenges, London, Edward Elgar Publishing.

[CR35] Grau, F. X., Escrigas, C., Goddard, J., Hall, B., Hazelkorn, E., Tandon, R. (Eds.) (2017). Towards a socially responsible higher education institution: Balancing the global with the local, GUNI Report. Girona.

[CR36] Hippel, E. (2005). *Democratizing innovation*. The MIT Press.

[CR37] International Standard ISO 26000. (2010). Guidance on social responsibility, ISO, Genève Switzerland. Assessed 30 January 2021 on: iso.org.

[CR38] Kahin, B., & Foray, D. (2006). *Advancing knowledge and the knowledge economy*. MIT Press.

[CR39] Klein, J. L. (2017). Social innovation, universities and the quest for social transformation. In *Towards a Socially Responsible Higher Education Institution: Balancing the Global with the Local, GUNI Report* (p. 165–180). Girona.

[CR40] McAdam, M., Miller, K., & McAdam, R. (2016). Situated regional university incubation: A multi-level stakeholder perspective. *Technovation*, *50–**51*, 69–78. 10.1016/j.technovation.2015.09.002

[CR41] Mihci, H. (2019). Is measuring social innovation a mission impossible? *Innovation: The European Journal of Social Science Research*, Vol 33 (3), 10.1080/13511610.2019.1705149

[CR42] Moulaert, F. (2009). Social innovation: Institutionally embedded, territorially (re)produced. In MacCallum, D., Moulaert, F., Hillier, J. & Vicari Haddock, S. (Eds), *Social innovation and territorial development*, Ashgate, Farnham, Surrey.

[CR43] Moulaert, F., Mehmood, A., MacCallum, D., & Leubold B. (2017). Social innovation as a trigger for transformations - The role of research, European Commission, Directorate-General for Research and Innovation, Available at http://ec.europa.eu/research/social-sciences/pdf/policy_reviews/social_innovation_trigger_for_transformations.pdf. Accessed at 16 November 2019

[CR44] Mulgan, G. (2010). Measuring social value, Stanford Social Innovation Review, https://ssir.org/articles/entry/measuring_social_value. Accessed 4 April 2018.

[CR45] Mulgan, G. (2007). Social innovation, what it is, why it matters and how it can be accelerated, The Young Foundation, London, Available at ResearchGate https://www.researchgate.net/publication/277873357_Social_Innovation_What_It_Is_Why_It_Matters_and_How_It_Can_Be_Accelerated. Accessed 5 April 2018

[CR46] Murray, R., Caulier-Grice, J., Mulgan, G. (2010). The open book of social innovation, The Young Foundation, NESTA, Available at NEST website https://media.nesta.org.uk/documents/the_open_book_of_social_innovation.pdf. Accessed 11 June 2018

[CR47] Neumeier, S. (2012). Why do social innovations in rural development matter and should they be considered more seriously in rural development research? –Proposal for a stronger focus on social innovations in rural development research. *Sociologia Ruralis, 52*(1), 10.1111/j.1467-9523.2011.00553.x

[CR48] Nowakowska, A. (2011), *Regionalny wymiar procesów innowacji*, Wydawnictwo Uniwersytetu Łódzkiego, Łódź.

[CR49] OECD – World Bank Institute. (2000). *Korea and the knowledge based economy. Making the transition*. Paris. Available at https://openknowledge.worldbank.org/handle/10986/13845. Accessed 17 March 2018

[CR50] OECD. (2000). A new economy? The changing role of innovation and information technology in growth. Paris. Available at http://www.oecd.org/sti/inno/aneweconomythechangingroleofinnovationandinformationtechnologyingrowth.htm. Accessed 19 March 2018

[CR51] Powell, W., & Snellman, K. (2004). The knowledge economy. *Annual Review of Sociology,**30*, 199–220. 10.1146/annurev.soc.29.010202.100037

[CR52] Puukka, J. (2017). Local and global engagement: Balancing needs at global, national and local level. In *Towards a Socially Responsible Higher Education Institution: Balancing the Global with the Local, GUNI Report* (p. 74). Girona.

[CR53] Radinger-Peer, V. (2019). What influences universities’ regional engagement? A multi-stakeholder perspective applying a Q-methodological approach. *Regional Studies, Regional Science,**6*(1), 170–185. 10.1080/21681376.2019.1578258 31565586 10.1080/21681376.2019.1578258 PMC6743734

[CR54] Reichert, S. (2006). The rise of knowledge regions: emerging opportunities and challenges for Universities. European University Association Publications, Brussles. Available at www.eu.be. Accessed 10 July 2018.

[CR55] Ripple, W. J., Wolf, Ch., Newsome, T. M., Galetti, M., Alamgir, M., Crist, E., Mahmoud, M. I., Laurance, W. F, 15,364 scientist signatories from 184 countries. (2017). *BioScience, 67*(12). 10.1093/biosci/bix125

[CR56] Riviezzo, A., Napolitano, M. R., Fusco, F. (2020). Along the pathway of university missions: A systematic literature review of performance indicators. In Daniel, A.D., Teixeira, A.A.C. & Preto, M.T. (Eds), *Examining the Role of Entrepreneurial Universities in Regional Development* (p. 24–50). Universidade de Lisboa, Portugal. 10.4018/978-1-7998-0174-0

[CR57] Roman, M., Varga, H., Cvijanovic, V., Reid, A. (2020). Quadruple Helix models for sustainable regional innovation: Engaging and facilitating civil society participation. *Economies 8(2)*10.3390/economies8020048

[CR58] Spaapen, J. van der Akker, W. (2017). *Productive interactions: societal impact of academic research in the knowledge society*. LERU Position paper. Available at file:///C:/Users/joann/Downloads/LERUPP_SocietalImpact_March2017.pdf. Accessed at 18 March 2021.

[CR59] Trencher, G. P., Yarime, M., & Kharrazi, A. (2013). Co-creating sustainability: Cross-sector university collaborations for driving sustainable urban transformations. *Journal of Cleaner Production,**50*, 40–55.

[CR60] Universities without walls. A vision for 2030. European Universities Association (2021). Report available on www.eua.eu. Accessed 4 February 2021.

[CR61] Vilalta, J.M., Jové, N., Gómez, V., Cayetano, M. (2020). 2nd GUNi International Conference on SDGs: Higher Education & Science Take Action. Barcelona. Available at http://www.guninetwork.org/. Accessed 11 October 2020.

[CR62] Wallace, M. (Ed.) (2017). Guidelines for Universities Engaging in Social Responsibility. Published by the UNIBILITY project. Available at ResearchGate. https://www.researchgate.net/publication/319910831_Guidelines_for_Universities_Engaging_in_Social_Responsibility. Accessed 16 February 2019.

